# Advances in New Alloys, Polymers and Composites for Biomedical Applications

**DOI:** 10.3390/ma19020304

**Published:** 2026-01-12

**Authors:** Javier Gil, Eugenio Velasco-Ortega, José Luis Rondón-Romero, Jesus Moreno-Muñoz, Enrique Nuñez-Marquez, Andreu Puigdollers, Marta Pegueroles

**Affiliations:** 1 Bioinspired Oral Biomaterials and Interfaces, Department Ciencia e Ingenieria de Materiales, Escola Enginyeria Barcelona Est., Universitat Politècnica de Catalunya, Av. Eduard Maristany 16, 08019 Barcelona, Spain; 2 Department of Stomatology, Faculty of Dentistry, University of Seville, c/Avicena s/n, 41009 Sevilla, Spain; evelasco@us.es (E.V.-O.); jolurr001@hotmail.com (J.L.R.-R.); je5us@hotmail.com (J.M.-M.); enrique_aracena@hotmail.com (E.N.-M.); 3 Department of Orthodontics, Universitat Internacional de Catalunya, 08195 Barcelona, Spain; andreup@uic.es; 4 Biomaterials, Biomechanics and Tissue Engineering Group (BBT), Department of Materials Science and Engineering, Barcelona Research Center in Multiscale Science and Engineering (CCEM), Institute for Research and Innovation in Health (IRIS), Universitat Politècnica de Catalunya (UPC), EEBE, 08019 Barcelona, Spain; marta.pegueroles@upc.edu; 5 Centro de Investigación Biomédica en Red-Bioingeniería, Biomateriales y Nanomedicina (CIBER-BBN), Instituto de Salud Carlos III, 28029 Madrid, Spain

The continuous evolution of biomedical technologies has been inextricably linked to advances in materials science. From early metallic implants to contemporary multifunctional, bioactive, and intelligent systems, materials have consistently played a pivotal role in enabling safer, more effective, and longer-lasting medical devices. The steady increase in global life expectancy, together with the rising prevalence of chronic diseases and traumatic injuries, has intensified the demand for advanced biomaterials capable of fulfilling increasingly complex mechanical, biological, and functional requirements.

Over recent decades, remarkable progress has been achieved in the development of metallic alloys, polymeric systems, and composite materials. These advances have been driven by breakthroughs in processing technologies, computational materials design, surface engineering, and an improved understanding of biological interactions across multiple length scales. As a result, the scope of biomedical applications has expanded substantially, encompassing load-bearing orthopedic implants, cardiovascular devices, tissue engineering scaffolds, drug delivery platforms, and bioelectronic interfaces. This editorial highlight key developments in emerging alloys, polymers, and composites for biomedical applications, with an emphasis on current challenges, evolving trends, and future opportunities. Rather than offering an exhaustive review, it provides a critical perspective on how these material classes are shaping the next generation of biomedical solutions.

Metallic materials have long served as the backbone of biomedical implants owing to their superior mechanical strength, fracture toughness, and fatigue resistance [1]. Stainless steels, cobalt–chromium alloys, and titanium-based alloys remain widely employed in orthopedic, dental, and cardiovascular applications. Nonetheless, conventional metallic systems often exhibit limitations associated with elastic modulus mismatch, corrosion behavior, and long-term biological response [2,3,4]. Contemporary research has therefore shifted toward tailoring alloy composition and microstructure to enhance mechanical compatibility with human tissues, mitigate adverse biological effects, and introduce new functionalities. This transition reflects a broader paradigm shift from inert structural materials toward biofunctional metallic systems.

Among metallic biomaterials, titanium and its alloys continue to dominate due to their excellent corrosion resistance and favorable biocompatibility. However, the relatively high elastic modulus of traditional alloys, such as Ti–6Al–4V, can induce stress shielding, ultimately leading to bone resorption and implant loosening [5,6]. To overcome this limitation, β-type titanium alloys incorporating elements such as niobium, tantalum, molybdenum, and zirconium have been extensively investigated. These alloys exhibit significantly reduced elastic moduli, approaching that of cortical bone, while maintaining adequate mechanical strength [7,8]. Importantly, they also avoid potentially toxic alloying elements, aligning with the growing emphasis on long-term biocompatibility. Complementary surface modification strategies—including anodization, laser texturing, and bioactive coatings—have further improved osseointegration and antibacterial performance. The synergistic combination of bulk alloy design and surface engineering has thus emerged as a powerful approach for optimizing implant performance [9].

Shape memory alloys (SMAs), particularly nickel–titanium (NiTi), have introduced unprecedented functionality into biomedical devices. Their capacity for large reversible deformations and superelastic behavior has enabled minimally invasive procedures and self-expanding devices, such as vascular stents, guidewires, and orthodontic appliances [10,11,12]. Despite these advantages, concerns regarding nickel ion release and potential hypersensitivity reactions persist. Ongoing research focuses on surface passivation, protective coatings, and the development of nickel-free SMAs to address these challenges. In parallel, advances in additive manufacturing have opened new avenues for fabricating complex SMA architectures with tailored properties [13].

One of the most transformative trends in metallic biomaterials is the development of biodegradable alloys designed to gradually dissolve in physiological environments after fulfilling their mechanical function. Magnesium-, iron-, and zinc-based alloys are at the forefront of this research. Magnesium alloys, in particular, are attractive due to their low density and elastic modulus comparable to that of natural bone [14]. However, their rapid corrosion rates pose challenges related to hydrogen evolution and the loss of mechanical integrity. Strategies including alloying, surface modification, and microstructural refinement are actively being pursued to control degradation kinetics. Biodegradable metals represent a paradigm shift in implant design, offering temporary mechanical support while eliminating the need for secondary removal surgeries and reducing long-term complications.

Polymers provide unparalleled versatility in biomedical applications owing to their tunable chemistry, mechanical properties, and degradation behavior. Both natural and synthetic polymers have been widely adopted in medical devices, tissue engineering, and pharmaceutical systems. Synthetic polymers such as poly (lactic acid), poly (glycolic acid), and their copolymers have become standard materials for resorbable sutures, fixation devices, and drug delivery systems, with degradation profiles that can be precisely engineered to meet specific clinical requirements [15,16].

Recent advances have increasingly emphasized the design of biomimetic polymers that replicate the structural and biochemical cues of native tissues. Rather than serving as passive supports, these materials are intended to actively interact with cells. By incorporating bioactive motifs, growth factors, or peptide sequences, polymeric scaffolds can direct cell adhesion, proliferation, and differentiation. Hydrogels represent a particularly prominent class of biomimetic polymers, especially suited for soft tissue applications. Their high-water content and viscoelastic properties closely resemble those of biological tissues, making them attractive for cartilage repair, wound healing, and cell encapsulation [17].

The emergence of stimuli-responsive polymers has further expanded the functional landscape of biomedical materials. These systems respond to external or internal triggers, such as temperature, pH, electric fields, or biochemical signals, enabling controlled and localized therapeutic action. Applications include targeted drug delivery systems that release therapeutic agents in response to pathological conditions, as well as soft actuators and sensors for implantable devices. The integration of such smart polymers represents a critical step toward adaptive and personalized medicine [18,19,20]. Increasingly, advanced polymer systems combine biodegradability with additional functionalities, including antimicrobial activity, electrical conductivity, and imaging capability, thereby addressing the growing demand for multifunctional biomedical devices.

Composite materials offer a unique strategy for integrating the advantageous properties of multiple constituent phases. In biomedical contexts, composites are engineered to overcome the limitations of single-material systems by achieving optimized mechanical performance, biological response, and functional integration. Hierarchical natural composites, such as bone and cartilage, have provided key inspiration for the development of biomimetic engineered counterparts [21].

Fiber-reinforced composites have attracted particular interest for load-bearing applications in orthopedics and prosthetics. Through careful control of fiber type, orientation, and volume fraction, these materials can be tailored to replicate the anisotropic mechanical behavior of natural tissues. Polymer matrix composites reinforced with bioresorbable fibers are especially promising for temporary implants, as they provide mechanical support during healing while progressively transferring load to regenerating tissue [22].

Nanocomposites, incorporating nanoscale fillers such as carbon nanotubes, graphene, or bioactive nanoparticles into polymeric or metallic matrices, have demonstrated substantial enhancements in mechanical strength, electrical conductivity, and biological performance, even at low filler contents. Such systems have shown considerable promise in antibacterial coatings, bone regeneration, and neural interfaces [23]. Nevertheless, the long-term safety and biological fate of nanomaterials remain critical areas of ongoing investigation.

In tissue engineering, composite scaffolds that combine biodegradable polymers with ceramics, bioactive glasses, or molecular cues enable the creation of environments conducive to tissue regeneration. These scaffolds can be designed with graded architectures and spatially varying properties, closely mimicking the structural complexity of native tissues [24]. Additive manufacturing techniques have further facilitated the fabrication of patient-specific composite scaffolds with controlled porosity and architecture.

Advances in additive manufacturing, computational modeling, and data-driven materials design have fundamentally transformed the development of biomedical materials. Three-dimensional printing enables unprecedented control over geometry and internal structure, supporting customization and rapid prototyping. Computational tools, including finite element analysis and machine learning, are increasingly employed to predict material behavior and optimize compositions prior to experimental validation, thereby accelerating innovation while reducing development costs [25].

Despite these advances, several challenges must still be addressed to ensure successful clinical translation, including long-term biocompatibility and degradation behavior, manufacturing scalability and reproducibility, the integration of multifunctionality without compromising safety, regulatory approval of complex material systems, and the development of durable bactericidal surfaces to prevent infection. Addressing these challenges will require close collaboration among materials scientists, clinicians, and regulatory authorities.

Looking forward, the future of biomedical materials lies in convergence: integrating materials science with biology, electronics, and data science. Emerging directions include biohybrid materials, self-healing systems, and intelligent implants capable of real-time monitoring and therapeutic intervention. Sustainability and ethical considerations are also expected to play an increasingly prominent role, guiding the development of materials that are not only effective but also responsible throughout their entire life cycle.

In conclusion, advances in alloys, polymers, and composite materials have profoundly reshaped the landscape of biomedical applications. Through innovative design, precise control of properties, and interdisciplinary collaboration, modern biomaterials are evolving from passive structural components into active and adaptive systems. Continued research and thoughtful translation will be essential to fully realize their potential in improving human health and quality of life.
